# Balance Disorders in People with History of COVID-19 in Light of Posturographic Tests

**DOI:** 10.3390/jcm12134461

**Published:** 2023-07-03

**Authors:** Zofia Dzięcioł-Anikiej, Agnieszka Dakowicz, Janusz Dzięcioł, Szymon Kopko, Diana Moskal-Jasińska, Aleksandra Gawlikowska-Sroka, Anna Kuryliszyn-Moskal, Amanda Maria Kostro

**Affiliations:** 1 Department of Rehabilitation, Faculty of Health Sciences, Medical University of Białystok, Skłodowskiej-Curie 7A Street, 15-096 Białystok, Poland; 2 Department of Human Anatomy, Faculty of Medicine, Medical University of Bialystok, Mickiewicza 2A Street, 15-230 Białystok, Poland; 3 Department of Clinical Phonoaudiology and Speech Therapy, Faculty of Health Sciences, Medical University of Białystok, Skłodowskiej-Curie 7A Street, 15-096 Białystok, Poland; 4 Department of Human Anatomy, Pomeranian Medical University in Szczecin, Rybacka 1 Street, 70-204 Szczecin, Poland

**Keywords:** COVD-19, posturography, balance, coronavirus, dizziness

## Abstract

Coronavirus disease-19 (COVID-19), resulting from infection with the SARS-CoV-2 virus, causes not only flu-like symptoms, such as fever, aches, or a dry cough, but also affects the sensory system, leading to a loss of smell and taste or to neurological deficits in the shape of balance disorders and dizziness. Purpose of the study: Our research aimed to assess the prevalence of balance disorders in patients who had suffered COVID-19. Material and methods: The study group consisted of 73 subjects with a history of SARS-CoV-2 infection. The control group consisted of 50 healthy people with similar demographics. A balance analysis was performed on a tensometric platform, using the Romberg test. Results: Statistically significant differences between the results of the study group and the control group were obtained in the evaluation of the length of body sways and the area of gravity center, both with open and closed eyes, and in the case of maximum body sways with open eyes. Conclusions: Patients who have suffered COVID-19 may suffer from balance disorders detectable by posturographic tests.

## 1. Introduction

COVID-19, the disease caused by the severe acute respiratory distress syndrome coronavirus 2 (SARS-CoV-2), presents with a range of symptoms, from mild ones (fever, aches and pains, dizziness, fatigue, or a dry cough) to grave ones (such as pneumonia or acute respiratory distress with cardiac sequelae) [1,2]. As reported by studies, the virus also affects the sensory system. The symptoms of an infection include smell and taste deficits, balance disorders, and neurotological problems [3,4,5]. 

Mao et al. were the first to report vertigo in the course of COVID-19 infections in 36 out of 214 hospitalized patients in 2020 [6]. Vertigo is classically vestibular and not a neurological symptom. Central vertigo can occur only if the vestibular nuclei pathways are affected. Another survey study (Violi et al.) comprised 185 patients, among whom 34 (18.4%) reported balance disorders following a diagnosis of COVID-19 [7]. Also, a study by Alde et al., conducted in 1512 patients (765 women, 747 men) aged 51 ± 18.4 years, confirmed the occurrence of dizziness in 251 (16.6%) patients, among whom 110 (43.8%) complained of lightheadedness, 70 (27.9%) of disequilibrium, 41 (16.3%) of presyncope, and 30 (12%) of vertigo. The results of this work suggest that vertigo should be included among the main symptoms of COVID-19, as one-sixth of patients reported this symptom, with women being significantly more affected than men (20.3 vs. 12.9%, *p* < 0.001) [8].

The Sia report shows that dizziness with accompanying gait instability can be considered an early symptom of SARS-CoV-2 infection [9]. In a systematic review of 14 studies and 141 patients, Saniasaya and Kulasegarah showed that vertigo was present in 4 to 30% of the cases studied [10]. A meta-analysis by Jafari et al. estimated that the incidence of dizziness in COVID-19 patients was 12.2% [11].

It should be emphasized that dizziness, lightheadedness, and balance disorders are usually caused by narcological and vestibular problems, but can also be caused by cardiac, ophthalmological, and proprioceptive problems. COVID-19 is known to affect multiple organ systems, including the cardiovascular and autonomic nervous systems. The systemic effects of the virus may contribute to the dysregulation of blood flow, heart rate, and other autonomic functions, potentially affecting those with POTS that results from COVID-19 [12]. 

It is recognized that the SARS-CoV-2 virus may exert a direct deleterious effect on neural structures, leading to encephalitis, neuron and nerve tissue damage, toxic infectious encephalopathy, or acute cerebrovascular disease. However, it is not fully established whether the virus causes dysfunction of the vestibular system or whether such dysfunction is a result of an ongoing infectious process within neural structures. Further research is needed, particularly to determine the cause of acute labyrinthitis, otitis media, or vestibular neuritis [10,13,14]. 

When analyzing balance, it is important to emphasize that it plays a crucial role in human life. For proper functioning, coordination of vestibular, ocular, and proprioceptive information by the central nervous system is essential. Several case reports and clinical observations provide reasons to speculate about the impact of SARS-CoV-2 coronavirus on the function of the balance system [15,16,17,18]. Patients most commonly indicate dizziness as the main symptom of balance disorders, but also complain of unsteadiness, postural instability, falls and trips, and non-vertiginous symptoms like basophobia and brain fog. [6,7,19,20]. Nevertheless, despite these reports, the assessment of balance in patients has usually been conducted in the form of questionnaires rather than through objective measurements. This means that there is still a lack of a comprehensive approach that would ensure an objective evaluation of the systems related to balance [10,21]. The latest publications already present methods of objective assessment of the occurring symptoms [22]. 

The aim of this study was to provide an objective assessment of the prevalence of balance disorders in patients after COVID-19 infection, measured by means of posturography analysis—the Romberg test—on a tensometric platform. 

## 2. Materials and Methods

The study was conducted at the Rehabilitation Clinic at the Medical University in Bialystok with the approval of the Bioethics Committee, no. APK.002.51.2022. Each patient signed a voluntary consent form to participate in the study and was informed about the consequences and the course of the study. 

The study group consisted of 73 people (42 women and 31 men) aged 24 to 75 (mean age 48), with an average BMI of 28.91. The tested participants obtained a positive result from a PCR test, confirming the presence of the virus. The studies were conducted 4–6 months after the end of treatment.

The control group consisted of 50 healthy people (29 women and 21 men) aged 22 to 72 (mean age 44), with an average BMI of 25.18. The study group was tested before the start of the COVID-19 pandemic, which confirmed that some people did not suffer from COVID-19 even asymptomatically.

The patients experienced a mild form of the disease, with the predominant symptoms being cough, fever, loss of smell, taste disorders, and myalgia. Excluded from the study were those with coexisting conditions which impaired balance, such as neuromuscular diseases, neural deficits, or sensory disorders, as well as those who had undergone spine or lower limb surgeries or had had injuries that significantly impaired stability (such as grade II-III ankle sprains), or those who relied on aids (crutches, canes, walkers, etc.) for daily activities. Previously, the presence of vestibular changes, biomechanical abnormalities of the lower limbs, including leg length discrepancies, and hyperelasticity were excluded. Ophthalmic problems were also ruled out. The control group comprised 50 people with a demographic structure similar to the study group. People from the control group did not have any ailments or neurological symptoms. The body mass index was determined for the patients in both groups. The characteristics of the studied groups are presented in Table 1. 

**Figure 1:**
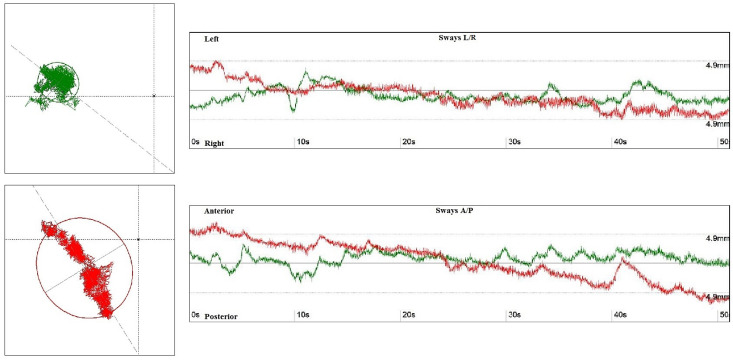
The surface area of the ellipse determined by the center of gravity with eyes open (green) and eyes closed (red)—left side. The graph shows the ranges of tilts in the anterior–posterior (A/P) and medial–lateral positions (L/P), with eyes open (green) and eyes closed (red)—right side.

Both patients and members of the control group underwent functional postural stability assessment on the FeeMED Base platform, using posturographic analysis with a 1-min Romberg test (Figure 1). 

The study of patients was performed by means of the same device in uniform environmental conditions, at the same time of day. Factor analyses were performed using the program Free Step v. 1.3.5. The posturographic assessment involved continuous assessment of the center of pressure (COP), which represents the displacement of the feet’s pressure center and allows for obtaining precise information about balance. The compared parameters included: length of sway (mm), surface area (mm^2^), weight on the right and left lower limb (%), speed (mm/s), and the magnitude of the minimum and maximum sways of the patient’s center of gravity in the successive tests with open and closed eyes. During the balance assessment, while standing on the platform, each subject was asked to focus their gaze on a designated point located on the wall, 1.5 meters in front of them. Comparisons between independent subgroups were conducted using the Mann–Whitney test, while comparisons before and after the pandemic were made using the Wilcoxon rank sum test. Linear regression models were used to estimate differences in balance assessments between groups, adjusted for age and BMI. All calculations were performed using IBM SPSS Statistics 27.0 software. Statistical hypotheses were tested at a significance level of *p* < 0.05.

## 3. Results

When comparing the control group to the study group, statistically significant results (*p* < 0.001) were obtained for: the length of sways with eyes open (Length of sways EO) and closed (Length of sways EC). 

Statistically significant results (*p* < 0.001) were obtained for: the surface area of the ellipse traced by the center of gravity with eyes open (surface area of ellipse EO) and closed (Surface area of ellipse EC) when comparing the control group and the study group.

For the speed of center of gravity movement within the base of support, no statistical significance was found when comparing the control group and the study group, both with eyes open and closed. 

As regards the parameters related to maximum sway, statistically significant differences between the control group and the study group were obtained in the evaluation of balance with eyes open, whereas for eyes closed, statistical significance was not achieved. Also, no statistical significance was found for the assessment of minimum sway in either open or closed-eye conditions. 

The characteristics of the tested parameters are presented in Table 2. 

In the assessment of the percentage load on the lower limbs, no statistically significant difference was found between the study and control groups in either closed or open-eye conditions for both the right and left lower limbs. 

The load values on the right foot were higher than on the left foot. The highest values occurred in patients from the COVID-19 group with their eyes open.

In the model that additionally takes into account age and BMI, the COVID group compared to the control group shows a significant difference in terms of the length of OO swings. This means that regardless of the influence of age and BMI, the COVID-19 group is different from the control group (Table 3). 

## 4. Discussion

Balance is a complex process that requires the integration of various stimuli to function properly. Also related to balance is stability, i.e., the ability to maintain and regain a state of equilibrium during or after performing a specific movement. The efficiency of the postural control system enables proper maintenance of static balance—during standing—as well as dynamic balance—during movement—despite the influence of external stimuli. This is expressed in the assessment of the center of feet pressure (COP) within the foot support area [23]. Balance disorders and dizziness are a set of non-specific symptoms associated with various etiology factors. Disorders of balance may also be related to various substrates that maintain balance, i.e., vestibular, ophthalmological, proprioceptive, and neurological contributions. Patients describe the presenting symptoms differently [24]. The most frequently reported are a feeling of dizziness, a sensation of movement in the head, instability, swaying, or spots in front of the eyes. The most common causes of dizziness include aging as well as complaints such as hypertension, sleep disorders, depression, anxiety, impaired motor function, or smoking. Dizziness can also occur as a side effect of a number of medications [25,26]. 

According to the latest reports, the diaphragm plays an important role in maintaining balance. This is due to its respiratory function, which supports the cardiovascular and lymphatic systems and provides adequate stability of the lumbar spine by creating pressure in the abdominal cavity. Studies suggest that after lung surgery, which results in a deterioration of diaphragm function, a worsening of balance parameters measureable by posturographic tests can be observed [27]. In patients infected with the SARS-CoV-2 virus, there is a deterioration in the functioning of the respiratory and muscular systems, also including the diaphragm, which may affect the ability to maintain balance. It is estimated that 80% of COVID-19 patients have diaphragm dysfunction, while 20% show atrophic changes within it. Corradi et al. showed that in COVID-19 patients, a thinner diaphragm was associated with a worse prognosis, including admission to the ICU and death. Lower thickness of the diaphragm decreases the strength of the respiratory pump [28,29,30]. 

The present study proved that balance in patients who had undergone COVID-19 was impaired in comparison with the persons from the control group. The results of the patients were worse as regards: length of sways of the gravity center and the surface area traced by it. 

In the study conducted by Guzik et al., which assessed 50 patients after mild COVID-19 infections compared to a control group using the AccuGait platform, it was demonstrated that individuals in the study group scored higher in the assessment of the length of the path and the mean speed of COP when tested with open eyes (*p* < 0.001). No differences were found between the groups in terms of any other measurements with eyes open. However, in the assessment of balance with closed eyes, the study group scored significantly higher in the assessment of the mean X (*p* = 0.022), ellipse area (*p* = 0.002), path length (*p* = 0.035), and mean speed (*p* = 0.026). It was concluded that a mild COVID-19 infection may cause balance disturbances in young adults [31]. 

In our study, significant differences were also found between the study group and the control group, particularly in parameters assessing the length of the path—sway length (*p* < 0.001)—with both open and closed eyes. We noted no statistically significant difference in the assessment of mean speed (*p* = 0.72) with closed eyes, but for the parameters of the area of the ellipse—which we refer to as surface area—we obtained statistical significance (*p* < 0.001). 

Another study (Yılmaz et al.) used Computer Dynamic Posturography (CDP), which allows for the determination of the contribution of sensory systems to postural control under dynamic or static conditions with closed and open eyes. The study found lower mean composite scores in the study group compared to the control group (*p* < 0.01); patients’ overall visual scores were also lower compared to the control group (*p* < 0.01). The researchers concluded that COVID-19 might cause dizziness, although not to a debilitating degree. It can be observed in every fifth adult after a SARS-CoV-2 infection. The resulting changes are likely irreversible, which may be confirmed by the fact that they persist after recovery [32]. 

This observation is in line with our findings, which suggest less balance control in patients after COVID-19 infection. 

El-Bagalaty et al. assessed 31 adults who had recovered from COVID-19 using the Berg scale and the biodex stability system (BSS). The SARS-CoV-2 group had significantly higher values for the anterior–posterior stability index (*p* = 0.013), medial–lateral stability index (*p* = 0.018), overall stability index (*p* = 0.011), and fall risk index (*p* = 0.008) compared to the control group. Additionally, the Berg balance scale results were statistically higher for the control group than for those who had been infected with the SARS-CoV-2 virus (*p* = 0.003) [33]. 

Gervasoni et al. performed a quantitative assessment of the occurrence of balance and proprioception deficits associated with post-COVID-19 syndrome in 66 outpatients, using the Hunova robotic device. Dynamic balance was measured with eyes open and closed, by means of three indicators: the path of sway and two ranges of oscillation, anterior–posterior and medial–lateral. Hospitalized patients had statistically significantly worse results compared to non-hospitalized patients for both the swaying path and range of oscillation, with the worst performance being observed in the test with closed eyes. These results suggest that balance deficits are associated with post-COVID-19 syndrome [34].

Also Giardini et al. assessed balance in COVID-19 patients and compared their results with those of patients with chronic obstructive pulmonary disease and healthy individuals. A total of 75 people were evaluated, both with eyes open and closed: 25 COVID-19 patients, 25 with chronic obstructive pulmonary disease, and 25 healthy individuals, using a stabilometric platform, as well as the Mini-BESTest and the timed up and go test to assess dynamic balance. The results showed that, in comparison with healthy individuals, patients after COVID-19 had worse static (*p* < 0.005) and dynamic (*p* < 0.0001) balance. Furthermore, the latter group showed similar results to patients with chronic obstructive pulmonary disease [35].

Similar results regarding static balance were obtained in the present study—patients in the study group had higher values of sway length and the surface area of the ellipse traced by the center of gravity, which confirms the hypothesis that post-COVID-19 patients experience balance disorders detectable by posturographic tests.

Furthermore, our study involved a precise and objective assessment of balance disorders, as it used equipment intended for posturographic evaluation, rather than a questionnaire or telephone survey method, which other studies have employed [36].

Zahra et al.’s meta-analysis and systematic literature review aimed to assess the frequency of hearing loss, tinnitus, and dizziness in patients after COVID-19. Their results suggest that hearing loss (3.1%, CI: 0.01–0.09), tinnitus (4.5%, CI: 0.012–0.153), and dizziness (12.2%, CI: 0.070–0.204) are statistically significant in patients with COVID-19 (Z ≤ −4.469, *p* ≤ 0.001). However, researchers caution against interpreting these results too literally, given insufficient evidence and the heterogeneity of existing studies. Moreover, they recommend further evaluation using standard objective tests [10]. Our study meets these requirements, because the equipment used made it possible to objectively evaluate balance disturbances by measuring the parameters suggesting a vestibular pathology.

## 5. Conclusions

The conducted study shows that people who have recovered from COVID-19 experience balance deficits, which can be confirmed by the Romberg test on a tensometric platform. The diagnostic method used in the study allows clinicians to quickly and accurately measure balance disorders and may serve as an excellent source of information enabling them to compare results before and after therapy.

## Figures and Tables

**Table 1:** Comparison of demographic data between the study groups.

Parameter	Control Group				COVID-19 Patients				Statistically Significant Difference
	N	Mean	Minimum	Maximum	N	Mean	Minimum	Maximum	*p* < 0.05
Age	50	44.02	21	74	73	48.55	27	75	0.000
BMI	50	25.18	18.44	38.37	73	28.91	20.88	41.03	0.000
	Gender								
Female	29 (58%)				42 (58%)				
Male	21 (42%)				31 (42%)				

**Table 2:** The characteristics of the tested parameters between COVID-19 patients and the control group.

		Control Group				COVID-19 Patients				
Parameter		Mean	SD	Min	Max	Mean	SD	Min	Max	*p*
Center of gravity sway length	EO	1331.13	433.91	292.73	2619.06	2368.88	744.47	830.53	4215.75	0.000
	EC	1409.41	466.65	333.90	2864.90	2330.27	764.57	1009.10	4764.99	0.000
Surface area of the ellipse traced by the center of gravity	EO	174.40	166.43	6.94	877.96	723.93	1276.05	34.06	7706.86	0.000
	EC	350.16	520.23	14.52	2642.9	578.18	512.73	51.74	2256.45	0.000
Speed of gravity center within the area of foot support	EO	36.02	12.47	5.72	62.17	38.91	12.26	13.61	70.94	0.358
	EC	38.32	12.79	6.55	61.14	38.29	12.52	16.5	78.08	0.720
Maximum sways	EO	1.68	0.69	0.96	5.86	2.23	2.20	0.92	18.81	0.001
	EC	2.05	1.,18	1	7.81	2.16	1.07	1.1	8.81	0.186
Minimum sways	EO	0.0050	0.0054	0	0.02	0.0032	0.0047	0	0.01	0.056
	EC	0.0058	0.0067	0	0.03	0.0042	0.0050	0	0.01	0.277
Load on the right lower limb	EO	52.92	5.63	42	63	53.16	8.36	25	76	0.819
	EC	53.60	6.52	36	66	51.92	8.37	16	71	0.127
Load on the left lower limb	EO	47.10	5.65	37	58	46.84	8.36	24	75	0.807
	EC	46.40	6.52	34	64	48.08	8.37	29	84	0.127

**Table 3:** Multivariable linear regression models adjusted for age and BMI.

Dependent Variable		Effect of COVID-19 Compared to Control Group			
		B	95% C.I.		*p*
Center of gravity sway length	EO	1329.510	1046.586	1612.435	0.000
	EC	1289.414	1003.569	1575.259	0.000
Surface area of the ellipse traced by the center of gravity	EO	439.740	−27.855	907.335	0.065
	EC	68.287	−174.229	310.803	0.581
Speed of gravity center within the area of foot support	EO	7.658	2.165	13.152	0.006
	EC	5.552	0.055	11.048	0.048
Maximum sways	EO	0.561	−0.273	1.395	0.187
	EC	0.188	−0.342	0.718	0.487
Minimum sways	EO	−0.002	−0.004	0.000	0.091
	EC	−0.001	−0.004	0.002	0.384
Load on the right lower limb	EO	−1.871	−5.322	1.581	0.288
	EC	−3.895	−7.469	−0.321	0.033
Load on the left lower limb	EO	1.861	−1.592	5.314	0.291
	EC	3.895	0.321	7.469	0.033

## Data Availability

The data presented in this study are available on request from the corresponding author (zofia.dzieciol-anikiej@umb.edu.pl).
